# MgO Nanoparticles as a Promising Photocatalyst towards Rhodamine B and Rhodamine 6G Degradation

**DOI:** 10.3390/molecules29184299

**Published:** 2024-09-11

**Authors:** Maria-Anna Gatou, Natalia Bovali, Nefeli Lagopati, Evangelia A. Pavlatou

**Affiliations:** 1 Laboratory of General Chemistry, School of Chemical Engineering, National Technical University of Athens, Zografou Campus, 15772 Athens, Greece; natalia.bovali@gmail.com; 2 Laboratory of Biology, Department of Basic Medical Sciences, Medical School, National and Kapodistrian University of Athens, 11527 Athens, Greece; nlagopati@med.uoa.gr; 3 Biomedical Research Foundation, Academy of Athens, 11527 Athens, Greece

**Keywords:** MgO, precipitation approach, photocatalysis, organic dyes, rhodamine B, rhodamine 6G, photocatalysis mechanism, photocatalyst selectivity, scavengers, reusability

## Abstract

The increasing global requirement for clean and safe drinking water has necessitated the development of efficient methods for the elimination of organic contaminants, especially dyes, from wastewater. This study reports the synthesis of magnesium oxide (MgO) nanoparticles via a simple precipitation approach and their thorough characterization using various techniques, including XRD, FT-IR, XPS, TGA, DLS, and FESEM. Synthesized MgO nanoparticles’ photocatalytic effectiveness was evaluated towards rhodamine B and rhodamine 6G degradation under both UV and visible light irradiation. The results indicated that the MgO nanoparticles possess a face-centered cubic structure with enhanced crystallinity and purity, as well as an average crystallite size of approximately 3.20 nm. The nanoparticles demonstrated a significant BET surface area (52 m^2^/g) and a bandgap value equal to 5.27 eV. Photocatalytic experiments indicated complete degradation of rhodamine B dye under UV light within 180 min and 83.23% degradation under visible light. For rhodamine 6G, the degradation efficiency was 92.62% under UV light and 38.71% under visible light, thus verifying the MgO catalyst’s selectivity towards degradation of rhodamine B dye. Also, reusability of MgO was investigated for five experimental photocatalytic trials with very promising results, mainly against rhodamine B. Scavenging experiments confirmed that •OH radicals were the major reactive oxygen species involved in the photodegradation procedure, unraveling the molecular mechanism of the photocatalytic efficiency of MgO.

## 1. Introduction

The rising global need for clean and safe drinking water is a direct consequence of water pollution, which also leads to epidemics in various countries [1]. Contaminated water is a major cause of widespread waterborne diseases [2]. Organic pollutants, including dyes, contribute to health problems such as cancer in both humans and animals. Additionally, water pollution has been linked to higher mortality rates [3].

Even though organic water pollution occurs through various industrial sources, including textile, pharmaceutical, papermaking, leather, printing, cosmetics, and food processing, the textile industry constitutes a significant factor as it contributes to the generation of a vast quantity of dye-containing wastewater, since it is estimated that annually ≈700,000 tons of dyes are produced, while due to inefficiencies in the dyeing process, around 200,000 tons of dyes are released into water bodies during dyeing and finishing operations. Dyes possess an aromatic molecular structure attributed to hydrocarbons, such as C_6_H_6_, C_6_H_5_CH_3_, C_14_H_10_, C_8_H_10_, C_10_H_8_, etc. [4,5]. In addition, they contain auxochromes (-NH_2_, -Cl, -OH, -COOH, etc.), as well as chromophores (carbonyl, azo, nitroso, nitro, sulfur functional groups, etc.). Chromophores, which receive electrons, provide color, while auxochromes, which donate electrons, enhance the adhesion and solubility of color on substrates. Many dyes dissolve in water, and even at concentrations below 1 ppm, these dyes color industrial wastewater, which reduces sunlight penetration into water bodies. This affects oxygen levels, hinders photosynthesis, and disrupts the balance of eutrophication processes [6,7].

Among numerous dyes, rhodamine B (RhB) and rhodamine 6G (R6G), which are being extensively used, pose a pivotal threat to aquatic ecosystems and human health. Rhodamine B is an aminoxanthene anionic dye, and it is acknowledged for its mutagenic, noxious, chemically inert, and non-biodegradable properties, making it particularly hazardous. In particular, it causes acute and chronic toxicity, while its accumulation within the body may potentially induce harm to the liver, kidneys, reproductive system, and nervous system, as well as promote carcinogenesis. Moreover, it can lead to allergies or skin irritation upon contact and, when inhaled, may cause coughing, shortness of breath, and chest pain [8]. Rhodamine 6G, also called rhodamine 590, is part of the xanthenes family and is commonly used in drug synthesis and in producing dyes like fluorescein and eosin. This dye constitutes a cationic polar compound with a stable heterocyclic structure, notable for its enhanced visible light absorption and intense fluorescence [9]. Rhodamine 6G is extensively utilized in dyeing materials such as acrylic, nylon, silk, and wool, while it constitutes the preferred dye towards dye laser applications and hydraulic flow pattern visualization, where it is utilized as a fluorescent tracer [10]. Additionally, R6G frequently serves as a sensitizer [11]. Recently, there has been an increasing focus on integrating R6G into both inorganic and organic matrices for use in areas like solid-state laser technology, optoelectronics, and optical filters [12,13,14]. Previous studies of our research group focusing on the degradation of various pollutants and dyes, such as methylene blue, methyl orange, brilliant green, etc., have shown that rhodamine is a very stable pollutant and is considered a very reliable system for a photocatalytic study; thus, it is selected also for this study, in parallel with the use of rhodamine 6G [15].

Currently, a range of standard treatment approaches is utilized, involving chemical precipitation, separation, adsorption, coagulation, reverse osmosis, ion exchange, flocculation, activated carbon adsorption, incineration, filtration, biopolymeric hybrid membrane technology, and electrochemical oxidation [16,17]. Nonetheless, these techniques frequently lead to incomplete dye degradation, generating secondary pollutants that require additional treatment and potentially exacerbating pollution [18,19]. To address these issues, there is rising interest in advanced oxidation processes (AOPs), which use semiconducting materials as an alternative to conventional approaches [20], offering various benefits, such as lower equipment demands, non-selective oxidation, straightforward control, cost-efficiency, and organic dyes’ complete conversion into harmless byproducts like CO_2_, H_2_O, other inorganic compounds, and/or less toxic organic compounds that are environmentally safe [21,22]. A distinguishing aspect of AOPs is their ability to produce reactive agents such as •OH, which enable the rapid and non-selective oxidation of organic pollutants. Particularly interesting is the use of heterogeneous photocatalysis with oxide-based nanomaterials, which effectively removes water-soluble organic contaminants from water/wastewater upon exposure to light [23].

Overall, in photocatalytic degradation, suspended particles in a water solution act as photocatalysts when exposed to light. In this process, the photocatalyst, which is typically composed of semiconductors with distinct electronic band structures characterized by a band gap (E_g_) separating the valence band (VB) and the conduction band (CB), plays a crucial role. The absorbance of photons, characterized by sufficient energy, leads to the generation of electron–hole (e^−^-h^+^) pairs within the semiconductor particles. Subsequently, these carriers undergo charge separation, promoting reactive species’ production such as H_2_O, •OH, and ^1^O_2_. It is important to note that the recombination of e^−^ and h^+^ does not require their participation in chemical reactions. The oxidative agents catalyze organic pollutants’ decomposition on or near the catalysts’ surface, eventually converting them into harmless substances [24].

Metal oxide semiconductors such as TiO_2_, ZnO, CuO, Fe_2_O_3_, Mn_2_O_3_, ZrO_2_, Co_3_O_4_, and WO_3_ exhibit outstanding adsorption properties and serve as effective catalysts because of their high reactivity, enhanced sensitivity to light, large surface area per unit mass, cost-effectiveness, non-toxicity, and enhanced catalytic performance in dye degradation through photodegradation [25].

Magnesium oxide (MgO) nanoparticles have attracted significant interest among metal oxide nanoparticles due to their excellent biocompatibility, non-toxicity, and strong stability under various conditions [26]. Additionally, the FDA considers MgO safe for human consumption [27]. MgO nanoparticles exhibit beneficial physicochemical properties, including increased ionic character, a significant specific surface area, unique crystal structures, and oxygen vacancies [28,29]. Nano-MgO particles can be fabricated utilizing a plethora of physicochemical techniques, such as sol–gel [30], microwave-assisted [31], solvothermal/hydrothermal [32], combustion [33], precipitation [34], environmentally friendly green synthesis [35], vapor deposition method [36], plasma irradiation [37], ultrasonic irradiation [38], etc. A variety of approaches have been employed to synthesize nano-MgO particles possessing decreased crystallite size and enhanced surface area, features that are acknowledged for augmenting photocatalytic performance towards organic dyes’ degradation upon irradiation [39,40] (Table 1). Among the utilized approaches, the precipitation method finds widespread application in synthesizing nanoparticles, as it is facile, cost-efficient, and useful for large-scale production [41].

In this study, MgO nanopowder was synthesized using a simple precipitation approach using Mg(NO_3_)_2_ (precursor) and NaOH as the precipitant. The physical characteristics of the nanopowder were comprehensively examined using techniques such as FESEM, XRD, FTIR, BET, DLS, and DRS. Following this, the photocatalytic efficiency of the material in degrading rhodamine B and rhodamine 6G was assessed under both UV and visible light, with a focus on its potential selectivity towards specific organic dyes. Additionally, the study aimed to elucidate the photocatalytic degradation mechanisms of the dyes under different light conditions, using scavengers during the experimental procedure to evaluate the oxidative potential of MgO nanoparticles that mediate the photocatalytic efficiency of this material.

## 2. Results

### 2.1. Characterization of MgO Powder

#### 2.1.1. XRD Analysis

**Figure 1:**
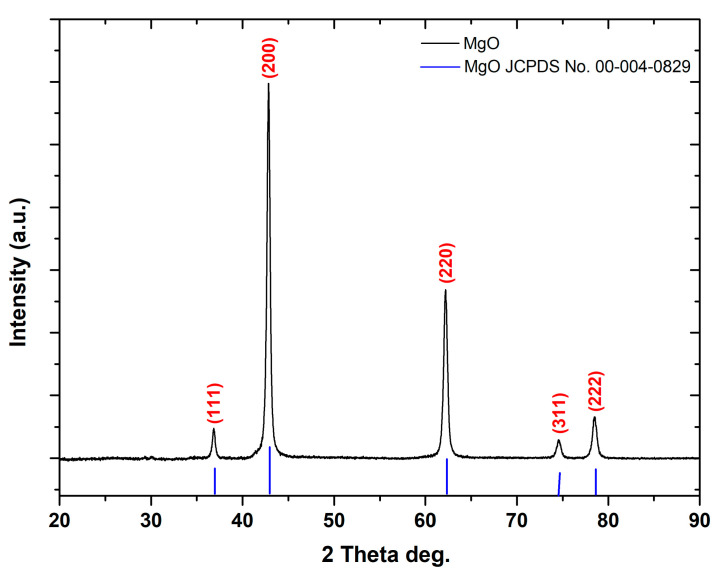
XRD diffractogram of the as-prepared MgO powder.

XRD was utilized in order to evaluate MgO powder’s crystallinity. The indexed peaks in the acquired pattern (Figure 1) are fully consistent with that of bulk MgO ((JCPDS) card no. 00-004-0829), certifying their monocrystallinity as well as face-centered cubic structure [48]. No additional impurity-related peak was spotted in the spectrum, within the detection limit of XRD, verifying the produced sample’s enhanced purity [49]. The formed peaks at two-theta (2θ) values, 36.85°, 42.83°, 61.20°, 74.58°, and 78.51°, are attributed to the (111), (200), (220), (311), and (222) (Miller indices) planes, respectively [50].

The as-produced MgO powder’s average crystallite size was determined through the Debye–Scherrer equation, its interplanar d-spacing according to Bragg’s Law Equation, and the crystallinity index (CI%), as previous studies have already analytically presented (Table 2 and Table 3) [51,52,53]. Bragg peak broadening (β) constitutes the composition of both the instrumental and sample-dependent effects. The instrumental peak width was corrected according to each diffraction peak of MgO material using the following equation (Equation (1)) [54]:

(1) $$\beta^{2}=\beta_{measured}^{2}-\beta_{instrumental}^{2}$$

The usual procedure towards instrumental broadening correction is determining the diffraction line breadth of a “coarse” material, such that broadening due to small crystallite size and lattice distortion is minimal. Thus, the “coarse” material chosen for reference was pure MgO (44–53 μm) and measured ten times in order to obtain statistical validity [55].

The value of the lattice constant was calculated using the following equation (Equation (2)), considering a cubic structure (*a* = *b* = *c*) (Table 2):

(2) $$d=\frac{a}{\sqrt{h^{2}+k^{2}+l^{2}}}$$

Additionally, the Nelson–Riley function (Equation (3)) was utilized for estimating the lattice constant due to its more enhanced precision in estimating lattice parameters after eradicating 2θ systematic errors for high angle reflections.

(3) $$F\left(\theta \right)=\frac{1}{2}\left(\frac{{cos}^{2}\theta }{\sin\theta }\right)+\left(\frac{{cos}^{2}\theta }{\theta }\right)$$

By extrapolating the lattice parameter’s straight line against an extrapolation function of θ to the value of 0 (Figure S1), the average lattice parameter (a) is determined. The acquired value aligns closely with the one reported in similar studies [56].

Thus, the lattice parameter (a) is measured equal to 4.2170 Å, which is marginally increased than the previously documented 4.2113 Å, according to the reference CIF (Crystallographic Information File) file [57]. Such a minor discrepancy in the lattice parameter is anticipated for nanoparticles possessing crystallite sizes in the tens of nanometers range.

In general, crystal imperfections and distortions lead to strain-induced broadening, which is expressed as ɛ ≈ β_s_/tan θ. A key aspect of Scherrer’s equation is its dependence on the diffraction angle θ. Unlike the Scherrer equation, which involves a 1/cos θ relationship, the Williamson–Hall approach shows variation with tan θ. This distinction is crucial because it enables the differentiation of reflection broadening when both small crystallite size and micro-strain co-exist. The following approaches consider that size and strain broadening are additive components of the total integral breadth of a Bragg peak [58]. The differing θ dependencies form the foundation for separating size and strain broadening in the W-H analysis. By combining the Scherrer equation with ɛ ≈ β_s_/tan θ, the following equations are obtained (Equations (4) and (5)):

(4) $$\beta_{hkl}=\beta_{s}+\beta_{D}$$

(5) $$\beta_{hkl}=\left(\frac{k\lambda }{D\cos\theta }\right)+(4\varepsilon \tan\theta )$$

where β_s_ refers to the broadening due to small crystallite size and β_D_ represents the broadening due to lattice distortions or micro-strain. The rearrangement of Equation (5) leads to the following equation (Equation (6)):

(6) $$\beta_{hkl}\cos\theta =\left(\frac{k\lambda }{D}\right)+\left(4\varepsilon \sin\theta \right)$$

where β_hkl_ constitutes the FWHM measured in radians, k equals to 0.9, λ corresponds to the wavelength of the X-rays (λ = 1.5406 Å), θ stands for the diffraction angle, D denotes the particle size, and ε constitutes the micro-strain [59]. Additionally, Equation (6) assumes that strain is uniform across all crystallographic directions, reflecting the isotropic nature of the crystal, where material properties do not vary based on the direction of measurement. A plot of βcos θ versus 4sin θ was made for the preferred orientation peaks of nano-MgO (Figure S2). In this plot, the slope corresponds to the strain, while the y-intercept indicates particle size. Typically, a negative slope indicates the presence of compressive micro-strain [60], whereas a positive slope suggests the possible presence of tensile micro-strain [59].

Based on the obtained results, the MgO powder presents a positive slope, thus affirming the existence of tensile micro-strain. In particular, the micro-strain within the sample was determined to be 2.16 × 10^−^^3^, indicating a small but noteworthy value, possibly attributed to the extremely small crystallite size of MgO, which was determined to be equal to 3.42 nm through the Williamson–Hall approach and equal to 3.23 nm applying the Scherrer approach. This small crystallite size prevents the relaxation of strain within the lattice [61]. Both for Scherrer and W-H calculations, zero shifts were accounted for by correcting 2θ.

#### 2.1.2. FT-IR Analysis

**Figure 2:**
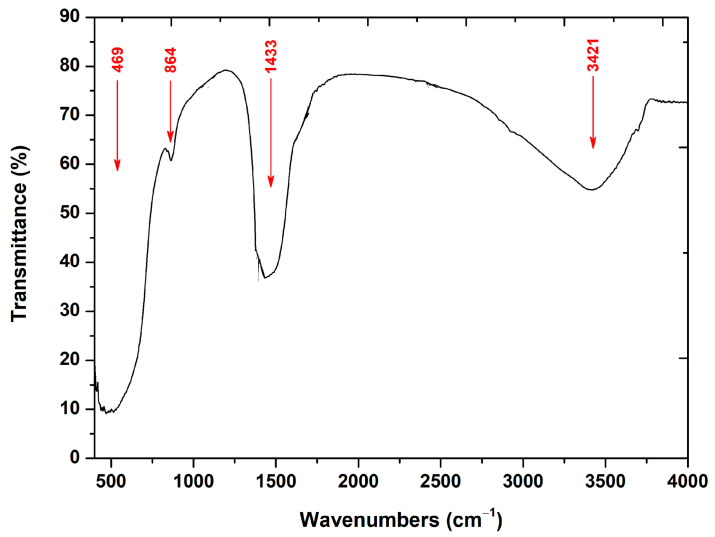
FT-IR spectrum of the synthesized MgO powder.

In the FT-IR spectrum of the studied MgO powder (Figure 2), bands at 468.62, 863.95, 1432.85, and 3421.10 cm^−^^1^ are illustrated.

In particular, the major band observed at ≈469 cm^−^^1^ is attributed to Mg-O vibrations [62]. The bands observed at approximately 864 and 1433 cm^−^^1^ are associated with carbonate species that are chemisorbed surficially on MgO [63], while the broad band depicted at 3421 cm^−^^1^ corresponds to the O-H stretching, as well as bending vibrations of H_2_O molecules [62,63], possibly due to atmospheric humidity during the conduction of the powder’s measurement [64].

#### 2.1.3. N_2_-Sorption Analysis

**Figure 3:**
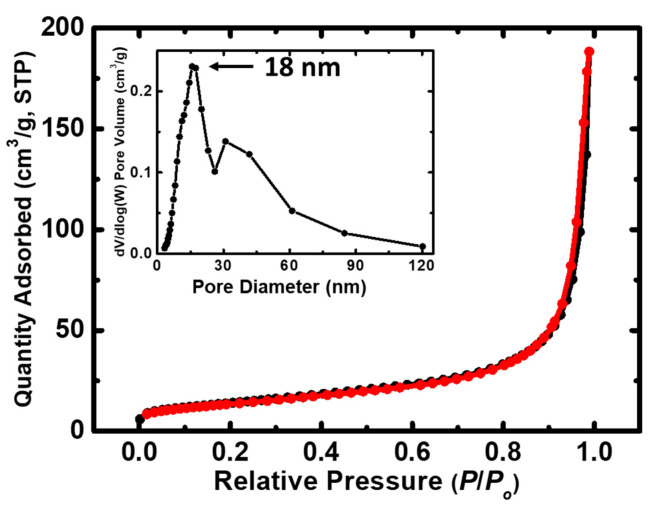
N_2_-sorption diagram of the prepared MgO powder (sorption: black line; desorption: red line). The pore size distribution utilizing the BJH approach is indicated in the inset.

The N_2_-sorption isotherm of the MgO powder is depicted in Figure 3.

Based on the acquired data, the as-synthesized powder displays a type IV isotherm, characterized by a narrow hysteresis loop and the absence of a saturation plateau, suggesting mesopores and macropores existence. The pore size distribution as obtained from the desorption curve via the BJH approach is depicted in the inset in Figure 3. This distribution is broad, covering both the mesopore range (2–50 nm) and the macropore range (>50 nm), consistent with the N_2_-sorption isotherm findings [65,66,67]. The physical parameters are summarized in Table 4, including the BET surface area, micropore surface area, cumulative volume, as well as average pore diameter. The prepared MgO powder exhibits an increased BET surface area, correlating with the small crystallite size, as observed through the XRD analysis (Table 2).

#### 2.1.4. XPS Analysis

**Figure 4:**
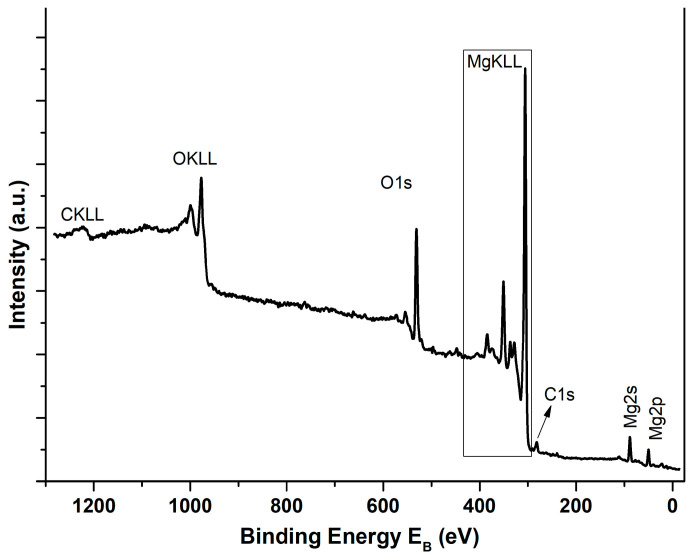
Wide survey XPS spectrum of the studied MgO powder.

**Figure 5:**
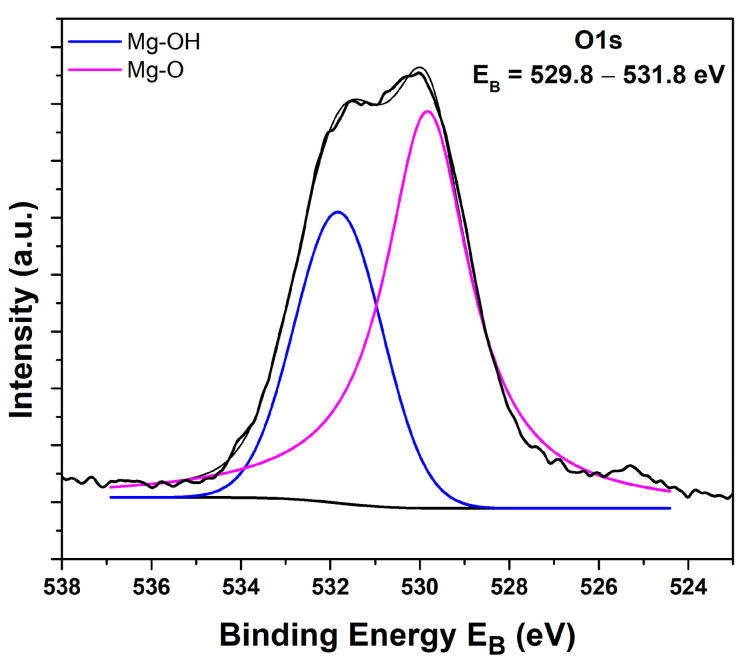
Deconvoluted O1s peak of the synthesized MgO powder.

XPS analysis was conducted to examine the prepared MgO powder’s surficial chemical composition. Figure 4 shows the wide survey spectrum of the as-synthesized powder. All peaks were expected due to the specific synthetic procedure that was employed. Figure S3a,b illustrates the detailed Mg2p XPS peak and the MgKLL X-ray-induced Auger spectrum (XAES). By adding the binding energy of Mg2p and the kinetic energy of MgKL_23_L_23_, the modified Auger parameter, which is an accurate method for chemical species characterization, is derived. The Mg2p binding energy was equal to 49.5 eV, and the modified Auger parameter was estimated as 1231.1 eV, both assigned to MgO [68]. Figure 5 indicates the deconvoluted O1s peak, which is a peak consisting of two components corresponding to oxides Mg-O (529.8 eV) and hydroxides Mg-OH (531.8 eV) [69]. The atomic percentage of Mg and O was calculated from the intensity (peak area) of the XPS peaks weighted with the corresponding relative sensitivity factors (RSF), taking into account the analyzer’s transmission characteristics, and was equal to 49.9% at. Mg and 50.1% at. O.

#### 2.1.5. TGA Analysis

**Figure 6:**
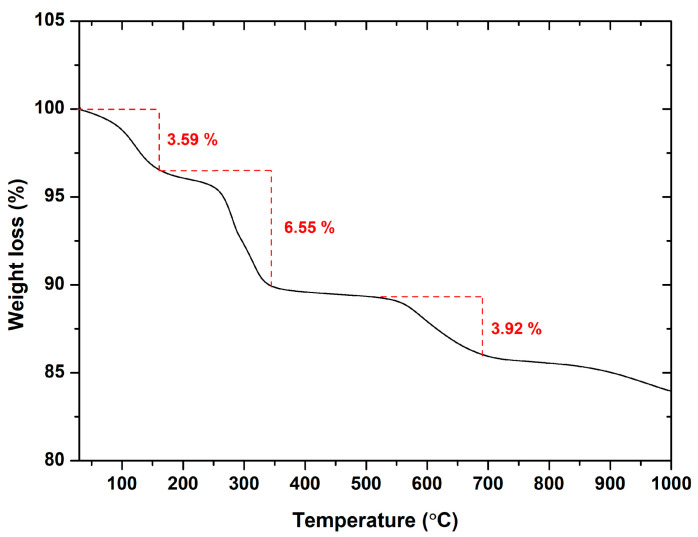
TGA spectrum of the as-prepared MgO powder.

The thermal stability of the developed MgO powder was investigated through thermogravimetric analysis by assessing weight loss, as depicted in Figure 6. Thermal decomposition occurred across three stages within the temperature range of 30–692 °C. The first stage, from 30 to 160 °C, resulted in 3.59% weight loss due to the evaporation of H_2_O and a minor amount of adsorbed CO_2_, probably due to prolonged storage [70]. During the second stage, between 165 and 345 °C, a 6.55% weight loss was noted, attributed to the decomposition of traces of Mg(OH)_2_ that have not been converted to MgO during calcination and organic residues’ oxidation, yielding carbon dioxide and water vapor. The third stage, ranging from 525 to 692 °C, led to a 3.92% weight loss due to carbonate decomposition and oxidation of remaining organic compounds. Above 692 °C, there was negligible weight loss, implying stabilization of the crystalline solid phases (magnesium hydroxide), as well as the enhanced thermal robustness of the synthesized MgO powder [71].

#### 2.1.6. Dynamic Light Scattering (DLS) Analysis

A crucial approach for characterizing nanoparticles is dynamic light scattering (DLS), which provides critical information about the size distribution of colloidal samples. It offers the ability to distinguish whether the studied nanoparticles are polydispersed (variation in size) or monodispersed (uniformity in size). Additionally, DLS analysis is instrumental in detecting aggregation or agglomeration that directly influences stability, reactivity, as well as efficacy of the examined nanostructure [72].

In the present study, the dynamic light scattering measurements were performed at a pH equal to 6.81 ± 0.01. Figure S4a depicts the distribution of hydrodynamic radius as a function of scattered light intensity of the studied MgO powder. Based on the acquired results, the as-utilized synthetic procedure yielded MgO possessing particle sizes within the range 10–100 nm and possessing an average particle size equal to ≈27 nm. The acquired value indicated the successful production of relatively small nanoparticles that are advantageous for photocatalytic applications. In general, smaller nanoparticles tend to exhibit enhanced stability in suspension and reduced aggregation or settling over time [73]. Moreover, decreased particle sizes offer increased surface area-to-volume ratios, potentially enhancing photocatalytic effectiveness [74]. In addition, the PDI (polydispersity index) value of the as-prepared MgO was equal to 0.197, confirming the uniform distribution of particle sizes as well as a monodisperse nature (PDI in the range 0–0.4) [75] (Table 5).

Furthermore, the zeta potential unveils important aspects of nanoparticles’ stability and behavior within a colloidal system [76]. Typically, dispersion systems characterized by zeta potential values ranging from ±0 to ±10 mV are considered highly unstable, while those between ±10 and ±20 mV are deemed stable. Furthermore, zeta potential values from ±20 to ±30 mV indicate moderately stable dispersions, and values exceeding ±30 mV indicate extremely stable dispersions [77]. The zeta potential of the as-synthesized MgO nanoparticles was measured equal to −50.8 mV (Figure S4b, Table 5), indicating their stability within the colloidal system. An enhanced absolute zeta potential value, particularly negative as observed from the obtained data, promotes strong repulsion among particles, thereby preventing agglomeration or precipitation over time.

#### 2.1.7. Diffuse Reflectance UV–Vis Spectroscopy (DRS) Analysis

Determining the energy band gap (E_g_) is essential for studies involving photocatalysis. Figure S5a presents the diffuse reflectance spectra (DRS) of the synthesized MgO powder.

To evaluate the powder’s reflectance, the Kubelka–Munk approach was utilized, as depicted in Figure S5a, following Equation (7) [78]:

(7) $$F\left(R\right)=\frac{{\left(1-R\right)}^{2}}{2R}$$

where R constitutes the reflectance.

As illustrated in Figure S5a, the absorption edge of the as-studied powder is located at ≈213 nm. Figure S5b depicts the direct energy band gap of the studied powder using the Kubelka–Munk model against energy through the extrapolation of the linear part of the spectra (F(R)hv)^1/2^ vs. hv. The E_g_ was determined utilizing Tauc’s equation (Equation (8)):

(8) $$ahv=A{\left(hv-E_{g}\right)}^{n}$$

where h constitutes the Planck’s constant, v stands for the frequency, α corresponds to the absorption coefficient, and n = ½ [53].

The studied MgO powder exhibited a band gap value equal to 5.27 eV. This finding is consistent with previous research, where E_g_ values for nano-MgO ranging from 5.0 to 6.2 eV were reported [79]. Additionally, the obtained energy band gap value is decreased, compared to the 7.8 eV reported for bulk MgO [80]. The reduced E_g_ value of the examined MgO powder could be attributed to its small crystallite size, as energy band gap narrowing may occur in the nano-scale region due to the high surface area to volume ratio of the crystallites [79]. This aspect is regarded as beneficial for enhancing the overall photocatalytic effectiveness of the as-prepared MgO powder [81].

#### 2.1.8. FESEM Analysis

**Figure 7:**
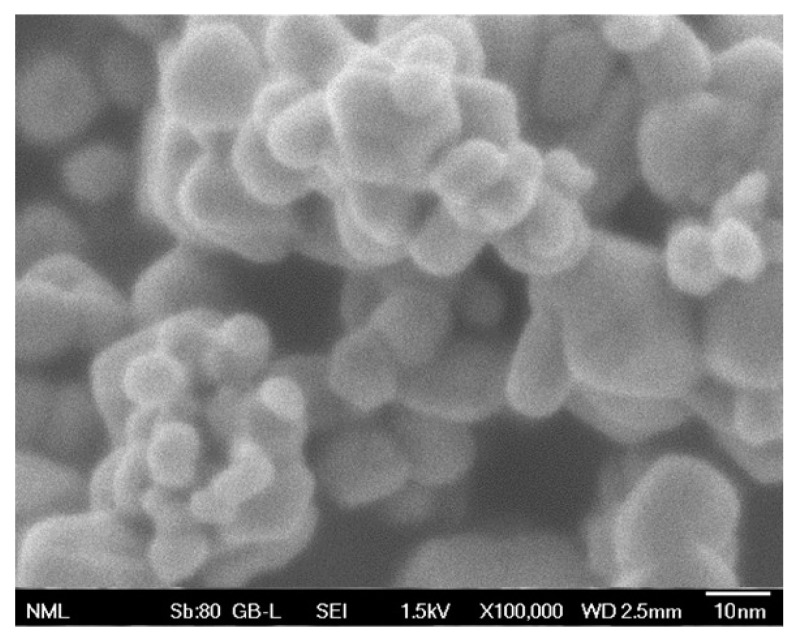
Representative FESEM image of the examined MgO powder at ×100,000 magnification.

The primary morphological characteristics of the synthesized MgO powder were assessed through FESEM observation, as illustrated in Figure 7.

Based on the obtained data, the observed nanoparticles display a combination of nearly spherical and hexagonal shapes, while they are also interconnected. This agglomeration might be attributed to electrostatic attraction, as well as polarity [82].

### 2.2. Photocatalytic Study of MgO Powder

#### 2.2.1. Study of the Photocatalytic Effectiveness towards Rhodamine B (RhB) Degradation

**Figure 8:**
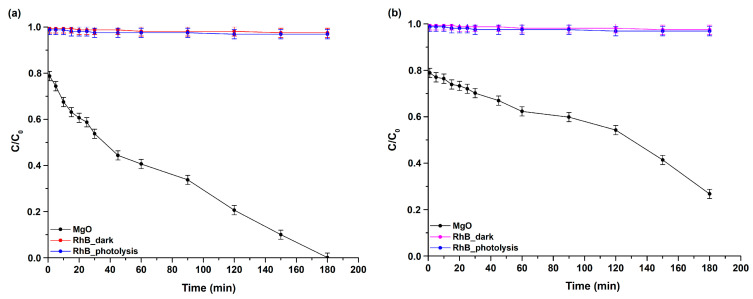
RhB’s degradation curve for the studied MgO powder vs. time upon (**a**) UV light exposure and (**b**) visible-light exposure. RhB’s photolysis and degradation under dark conditions are also included.

The photocatalytic capability of the as-developed MgO powder was primarily assessed by evaluating its efficiency towards RhB degradation within an aqueous solution under both visible and UV light illumination. The trials were carried out at room temperature and pH = 6.71 ± 0.01. Figure 8a,b illustrates the photocatalytic performance of MgO powder upon UV and visible light irradiation, respectively. Control experiments included photolysis (RhB photolysis) and adsorption–desorption equilibrium (RhB dark) in the absence of irradiation but with constant stirring for the same duration as the photocatalytic trials. The findings revealed that ≈3% of RhB degraded under both visible and UV light exposure, indicating an extremely low degradation rate of RhB in the absence of the examined powder. Moreover, consistent results from the trials implemented under dark conditions confirmed the dye’s robustness [83].

According to the received data, during the photocatalytic experiments, the examined powder exhibited high efficiency, as it led to RhB’s complete degradation (100%) within 180 min upon UV light illumination, as well as 83.23 ± 0.83% under visible light exposure within the same 180 min timeframe. Additionally, Figure S6a,b illustrates the UV–visible spectra documented throughout the photocatalytic trials, which were used to track the dye’s degradation progress over time, analyze the underlying degradation mechanisms, and evaluate the photocatalytic performance of the powder. Generally, RhB degradation proceeds through two known pathways: (a) N-de-ethylation and (b) disruption of its conjugated structure. Pathway (a) is characterized by a blue shift in the absorption maximum, while the pathway (b) shows a gradual decrease in absorption without a significant blue shift [84]. The real-time UV–visible spectra obtained during the photocatalytic trials of the MgO powder under UV (Figure S6a) and visible light (Figure S6b) clearly demonstrate the involvement of the second pathway during RhB’s degradation.

For the confirmation of the results obtained from RhB’s photocatalytic degradation, further analysis was conducted via TOC measurements so as to determine the percentage of mineralization of the examined dye attained during the photocatalytic process. RhB’s mineralization percentage was estimated via Equation (9):

(9) $$Mineralization\;\left(\%\right)=\left(1-\frac{{TOC}_{final}}{{TOC}_{initial}}\right)\times 100$$

where TOC_initial_ refers to the medium’s initial total organic carbon concentration prior to photocatalytic trials, while TOC_final_ denotes the medium’s total organic carbon concentration upon the completion of the photocatalytic procedure [85]. According to the TOC analysis, the MgO powder demonstrated almost total mineralization (98.83 ± 0.97%) of RhB dye upon UV light exposure, as well as an increased mineralization rate (80.04 ± 1.13%) under visible light illumination, thus validating the data acquired from RhB’s degradation study.

##### Study of RhB’s Photocatalytic Degradation Kinetics

**Figure 9:**
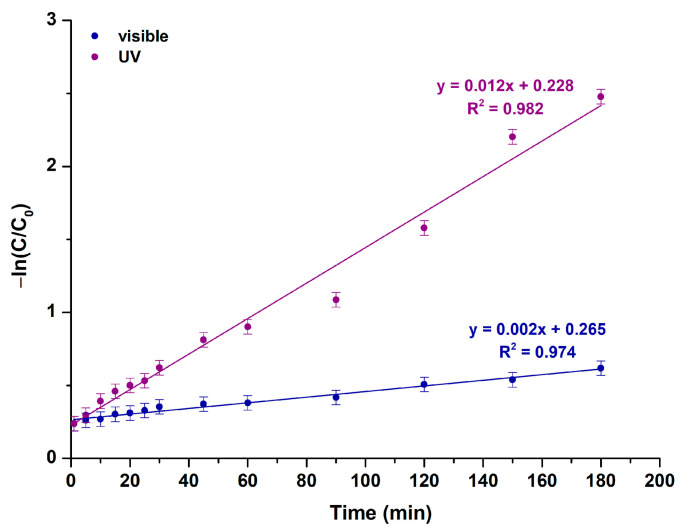
Photocatalytic kinetic model studies for the studied MgO powder, based on a pseudo-first-order model upon UV and visible light illumination.

Figure 9 indicates the outcomes derived from the investigation utilizing the pseudo-first-order kinetic model upon UV and visible light exposure, presenting a plot of −ln(C/C_0_) against time, as described by Equation (10) [86]:

(10) $$-ln\left(\frac{C}{C_{0}}\right)=k_{1}t$$

where C_0_ and C are ascribed to the initial and reaction-time RhB concentrations, respectively, k_1_ constitutes the photocatalytic oxidation’s apparent rate constant (min^−1^), while t stands for the irradiation time. The apparent rate constants of the as-prepared MgO powder under both types of irradiation derive from the linearly fitted plot’s slope.

However, the photocatalytic kinetics can alternatively be described by the pseudo-second-order equation (Equation (11)) [53]:

(11) $$\frac{t}{q_{t}}=\frac{1}{k_{2}q_{e}^{2}}+\frac{1}{q_{e}}t$$

where q_t_ and q_e_ constitute the amount of the pollutant adsorbed at time t and equilibrium, respectively (mg/g), and k_2_ corresponds to the rate constant (g/mg·min).

In contrast to the results observed with the pseudo-first-order kinetics (Figure 9), the R^2^ values acquired from the pseudo-second-order kinetic model (Figure S7) indicate a considerably decreased goodness of fit [53]. Table 6 details the kinetic parameters for the examined MgO powder.

In photocatalytic systems, rate constants are strongly influenced by crystallite size and specific surface area, both of which play critical roles in determining photocatalytic efficiency. A smaller crystallite size generally leads to a higher surface-to-volume ratio, which increases the number of active sites available for catalytic reactions, thereby enhancing overall performance [87]. However, this variability in surface area complicates direct comparisons between different photocatalysts, as larger specific surface areas may artificially boost rate constants by providing more reaction sites without necessarily improving the material’s intrinsic photocatalytic ability [88]. For instance, studies have shown that photocatalysts with larger surface areas often exhibit higher degradation efficiencies, due to increased dye adsorption (dye sensitization) rather than enhanced photocatalytic mechanisms [89]. As such, comparing photocatalysts with different specific surface areas may result in misleading conclusions about their relative efficiencies. In this study, the MgO nanoparticles demonstrated a surface area of 52 m^2^/g, which likely contributes to their observed photocatalytic performance.

##### Mechanism Study

During the photocatalytic oxidation procedure, several key oxidative species play a crucial role, including superoxide radicals (●O_2_^−^), singlet oxygen (^1^O_2_), electrons (e^−^), holes (h^+^), as well as hydroxyl radicals (●OH). In order to better understand the underlying photocatalytic mechanism, extensive studies were carried out to specify the active species. This involved a series of scavenging experiments in order to identify these specific species. In particular, p-benzoquinone (p-BQ, C_6_H_4_(=O)_2_, ≥98%, Sigma-Aldrich, Darmstadt, Germany), sodium azide (NaN_3_, ≥99.5%, Sigma-Aldrich, Darmstadt, Germany), silver nitrate (AgNO_3_, >99%, Sigma-Aldrich, Darmstadt, Germany), disodium ethylenediaminetetraacetate (EDTA-2Na, C_10_H_14_N_2_Na_2_O_8_●2H_2_O, ≥97%, Sigma-Aldrich, Darmstadt, Germany), and t-butanol (t-BuOH, (CH_3_)_3_COH, ≥99.5%, Sigma-Aldrich, Darmstadt, Germany) were added to the RhB dye’s solution, in order to selectively capture the ●O_2_^−^, ^1^O_2_, e^−^, h^+^, and ●OH, respectively [90,91].

**Figure 10:**
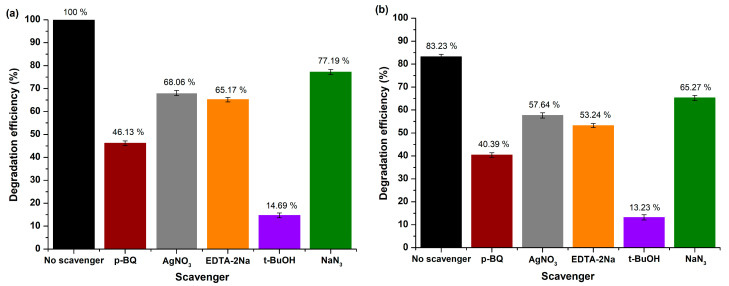
Scavenging trials for RhB’s degradation over MgO powder under (**a**) UV and (**b**) visible light exposure.

Derived from the outcomes depicted in Figure 10a,b, rhodamine B’s degradation effectiveness on the surface of the MgO powder endured a prominent reduction to 14.69 ± 1.03% and 13.23 ± 1.11% under UV and visible light illumination, respectively, upon adding t-BuOH into the photocatalytic reaction solution, thus confirming that the ●OH radicals had a major effect on RhB’s photocatalytic degradation in both irradiation conditions. On the contrary, ●O_2_^−^, ^1^O_2_, as well as photogenerated e^−^ and h^+^, were not the principal reactive species participating in the process.

**Figure 11:**
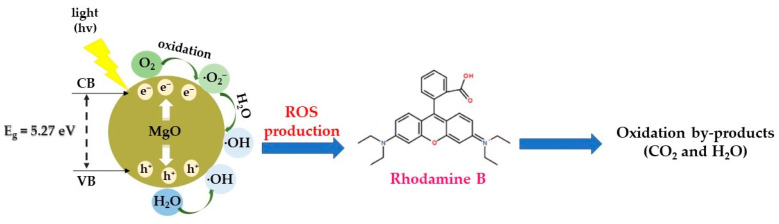
Proposed photocatalytic mechanism of MgO powder towards RhB degradation under both UV and visible light exposure.

In accordance with the results of the scavenging experiments, a feasible mechanism is outlined (Figure 11). When MgO nanoparticles are exposed to light (UV or visible) in the VB and CB, electrons and holes are produced within the reaction medium. Then, these photogenerated e^−^ interact surficially with the photocatalyst, leading to the oxidation of O_2_ to •O_2_^−^, while the photogenerated h^+^ tend to reduce -OH groups deriving from H_2_O molecules to •OH radicals. Subsequently, a reaction among •O_2_^−^ and H_2_O leads to the generation of -OH and HOO• radicals, which in turn produce •OH radicals. These free radicals facilitate the decomposition of RhB dye into both gaseous and liquid oxidation byproducts such as CO_2_ and H_2_O. The following equations illustrate the procedure of radical generation and demonstrate that •OH radicals are predominantly in charge of RhB’s degradation (Equations (12)–(18)):

(12) $$MgO+hv\to h_{VB}^{+}+e_{CB}^{-}$$

(13) $$H_{2}O\to H^{+}+-OH$$

(14) $$h_{VB}^{+}+-OH\to •OH$$

(15) $$e_{CB}^{-}+O_{2}\to •O_{2}^{-}$$

(16) $${•O}_{2}^{-}+H_{2}O\to HOO•+-OH$$

(17) $$HOO•+-OH\to 2•OH+O_{2}$$

(18) $$•OH+RhB\to oxidation\;byproducts+{CO}_{2}+H_{2}O$$

##### Reusability Study

**Figure 12:**
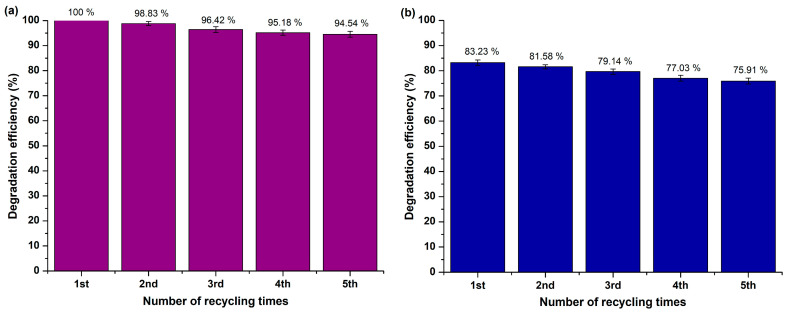
Reusability effectiveness of MgO powder after five experimental photocatalytic trials towards RhB degradation upon (**a**) UV and (**b**) visible light illumination.

Figure 12a,b demonstrates the reusability of the MgO nanopowder under both UV and visible light exposure across five successive photocatalytic cycles (catalyst loading = 5 mg, pH = 6.71 ± 0.01, initial concentration of RhB = 10 mg/L). After each degradation cycle, the photocatalyst underwent centrifugation and multiple washes with distilled H_2_O, followed by drying in a vacuum oven (70 °C, 24 h) in preparation for the next trial, with no further treatment [92]. The photocatalyst showed significant photostability under both light sources, as an approximate 5% (5.46 ± 0.83%) (Figure 12a) and a ≈7% (7.32 ± 1.01%) (Figure 12b) decrease in its photocatalytic efficiency was observed in the case of UV and visible light irradiation, respectively, after five consecutive cycles. These results verify the robustness of the examined photocatalyst throughout repeated photocatalytic trials.

Additionally, the studied powder was examined for its stability after five experimental cycles under the as-mentioned conditions through XRD (Figure S8). The analysis revealed that the MgO powder indicated insignificant changes in their crystalline phases, with only a slight increase in peaks’ intensity, proving that the examined photocatalyst maintained its structure after RhB’s degradation trials and exposure to air, presenting enhanced photochemical robustness. Moreover, the modest augmentation in peaks’ intensity may be attributed to crystallite size’s growth, because of the photoirradiation activation procedure [93].

#### 2.2.2. Study of the Photocatalytic Effectiveness towards Rhodamine 6G (R6G) Degradation

**Figure 13:**
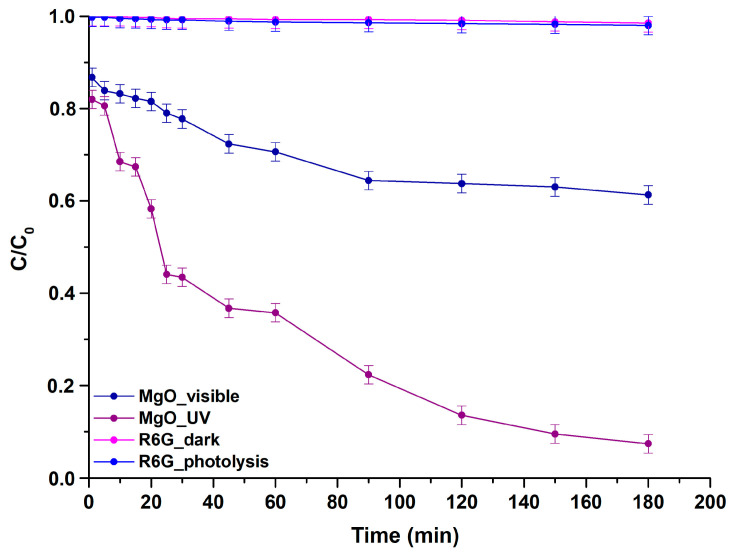
Degradation curves of R6G for the studied MgO powder vs. time upon UV and visible light exposure. R6G’s photolysis and degradation under dark conditions are also included.

MgO’s capability was also evaluated towards R6G’s (aqueous solution) photocatalytic degradation under the same irradiation conditions as the ones described in the case of rhodamine B. During R6G’s photocatalytic trials, temperature and pH conditions were set at 25 °C and 7.48 ± 0.01. Control trials were also conducted, including photolysis (R6G photolysis) and adsorption–desorption equilibrium (R6G dark) in the absence of light illumination upon continual stirring for the same duration as the photocatalysis procedure. The data acquired from these trials and for both irradiation types, verified the dye’s robustness, as ≈2% of R6G was degraded [86] (Figure 13).

Throughout the photocatalytic trials, the studied powder demonstrated enhanced effectiveness towards R6G degradation under UV light illumination, achieving a degradation rate equal to 92.62 ± 0.84% over 180 min, whereas a rate of the order of 38.71 ± 1.43% was attained upon visible light irradiation within the same period.

**Figure 14:**
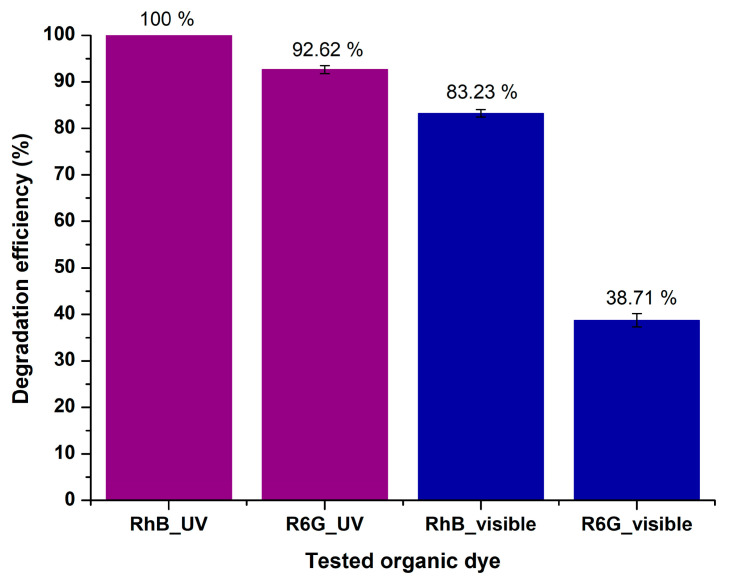
Selectivity of MgO photocatalyst.

Based on the data derived from the photocatalytic effectiveness studies for RhB, as well as R6G, the MgO powder achieved 100% and 83.23 ± 0.83% RhB degradation upon UV and visible light irradiation within 180 min, respectively, while 92.62 ± 0.84% and 38.71 ± 1.43% of R6G was degraded in the same timeframe upon UV and visible light illumination, respectively. Consequently, the as-mentioned photocatalyst exhibits selective activity favoring RhB’s photocatalytic degradation, primarily in the case of visible light irradiation (Figure 14). This phenomenon might be attributed to the pH that was prevalent during the experimental procedure. According to other studies, rhodamine B can be effectively degraded in generally acidic conditions, while rhodamine 6G requires highly basic conditions [94]. In these series of experiments, pH was approximately 7 (in the case of rhodamine B, pH was 6.71, and for rhodamine 6G, pH was 7.48). It might be possible to obtain even more promising results for rhodamine 6G for a pH of around 10.

Figure S9a,b represents the real-time UV–visible spectra as received during the photocatalytic trials. In general, R6G dye contains a chromophore made up of benzene and xanthene rings, connected by ethylamine (CH_3_CH_2_NH_2_) as the auxochrome. The chromophore determines the dye’s color, while the auxochrome influences the color’s intensity. The photocatalytic degradation of R6G typically follows two main pathways: breaking the conjugated chromophores or *N*-deethylation of the auxochromes. Previous research has outlined that a shift to a shorter absorption wavelength (blue shift) indicates a degradation pathway via *N*-deethylation [95]. Based on the emerged spectra, the peak at 526 nm (absorption maximum), which is attributed to a xanthene compound [96], remains constant, presenting no significant blue or red shift, thus rendering the *N*-deethylation pathway less probable in R6G’s degradation.

TOC analysis was similarly conducted to assess the extent of R6G’s mineralization (Equation (9)) during photocatalysis, so as to affirm the validity of degradation experiments. The as-mentioned analysis indicated that the MgO powder achieved a more increased mineralization rate of R6G upon UV light illumination (90.03 ± 1.31% instead of 36.49 ± 1.14% in the case of visible light irradiation), thus validating the results obtained from the photocatalytic degradation study.

##### Study of R6G’s Photocatalytic Degradation Kinetics

**Figure 15:**
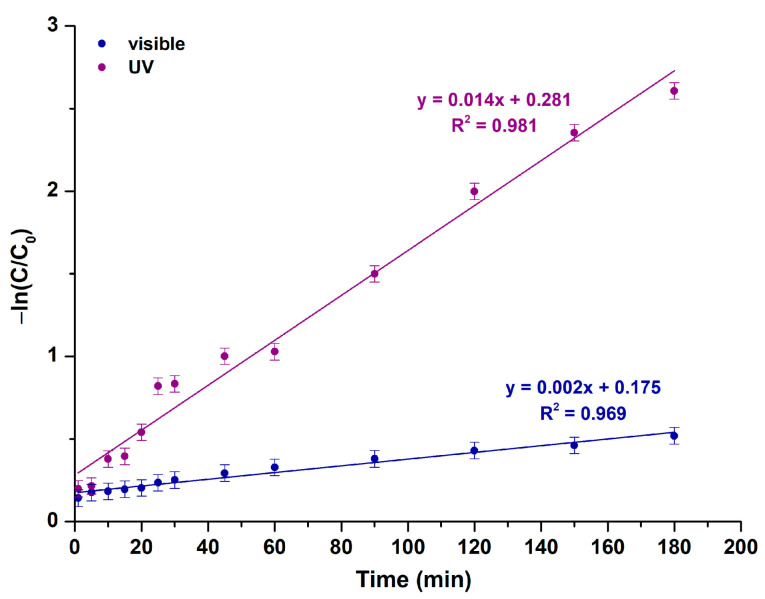
Photocatalytic kinetic model studies for the examined MgO powder, based on a pseudo-first-order model upon UV and visible light illumination.

Kinetic model studies upon UV and visible light illumination were conducted based on the pseudo-first-order (Equation (10) and Figure 15) and pseudo-second-order (Equation (11) and Figure S10) models.

In opposition to the pseudo-first-order model, the pseudo-second-order is characterized by inferior R^2^ values (Table 7). Consequently, it can be inferred that the photocatalytic degradation of R6G in the presence of the as-synthesized MgO powder upon both UV and visible light illumination is best elucidated by a pseudo-first-order reaction kinetic model.

##### Mechanism Study

**Figure 16:**
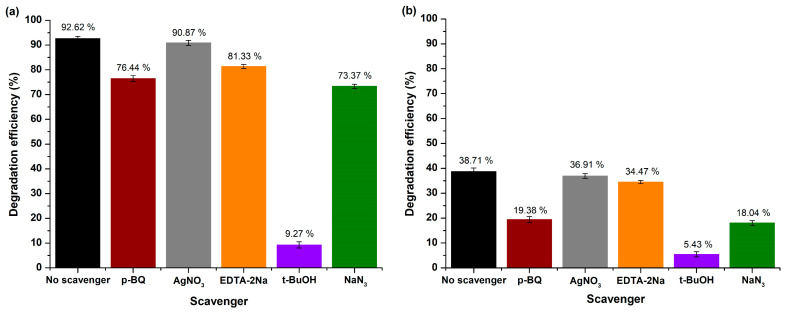
Scavenging trials for R6G’s degradation over MgO powder under (**a**) UV and (**b**) visible light exposure.

Comprehensive studies were conducted to determine the active species involved by emphasizing validating R6G’s photocatalytic degradation mechanism. Similar to the approach outlined in the case of RhB dye, experimental trials were performed to scavenge and capture the entagled active species. Consequently, AgNO_3_, EDTA-2Na, p-BQ, NaN_3_, and t-BuOH were added to R6G’s aqueous solution to selectively trap, as well as specify e^−^, h^+^, ●O_2_^−^ radicals, ^1^O_2_, and ●OH radicals, respectively (Figure 16a,b).

According to the received data, R6G’s capability presented a notable reduction to 9.27 ± 1.23% and 5.43 ± 1.01% under UV and visible light photocatalysis, respectively, after introducting t-BuOH into the photocatalytic reaction solution, thus justifying that ●OH radicals played a crucial role on R6G’s degradation in both irradiation conditions. However, when visible light was utilized as the source of irradiation, less oxidative species like •O_2_^−^ radicals and ^1^O_2_ indicated a slightly enhanced contribution to the degradation of R6G, possibly because under visible light the mechanism of self-sensitization was involved in the dye’s degradation [92]. Additionally, in both irradiation conditions, h^+^ had a minor effect on the degradation procedure, while the role of photogenerated e^−^ was negligible, proving the efficient e^−^ transfer from MgO’s surface towards the adsorbed molecules for the generation of reactive species [97].

As a result, taking also into account the as-received real-time UV–visible data, the suggested mechanism involves the cleavage of conjugated chromophores, where the predominant •OH radicals fragment R6G chromophore’s structural ring, leading to the effective dye’s degradation into mineralized by-products (CO_2_ and H_2_O).

##### Reusability Study

**Figure 17:**
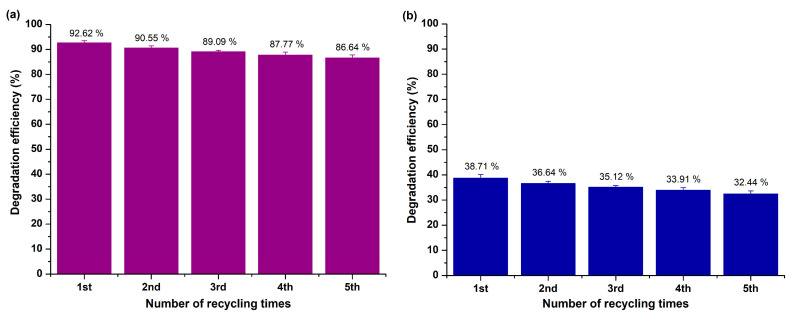
Reusability effectiveness of MgO powder after five experimental photocatalytic trials towards R6G degradation upon (**a**) UV and (**b**) visible light illumination.

The reusability of the studied nano-MgO powder upon both UV and visible light illumination across five sequential photocatalytic cycles (catalyst loading = 5 mg, pH = 7.48 ± 0.01, R6G’s initial concentration equal to 10 mg/L) was assessed (Figure 17a,b) through the perpetual process as in the case of RhB. The examined photocatalyst presented notable photostability under both utilized light sources, achieving a ≈6% decrease in its photocatalytic efficiency upon UV (5.98 ± 0.54%) and visible (6.27 ± 0.71%) light irradiation after the completion of the reusability experimental trials.

## 3. Discussion

This study successfully synthesized MgO nanoparticles using a simple precipitation method and evaluated their photocatalytic efficiency in degrading rhodamine B (RhB) and rhodamine 6G (R6G) upon UV and visible light illumination. The characterization of MgO nanoparticles confirmed their crystallinity, purity, and favorable surface properties, which are crucial for photocatalytic applications.

The XRD analysis revealed that the synthesized MgO nanoparticles possess a pure face-centered cubic structure with high crystallinity that is known to enhance photocatalytic activity towards degradation of organic dyes, indicating successful synthesis without significant impurities. The average crystallite size, determined using the Debye–Scherrer equation, was approximately 3.23 nm, which is beneficial for enhancing photocatalytic activity due to the increased surface area-to-volume ratio.

FT-IR analysis further confirmed the presence of characteristic Mg-O vibrations and minor surface-adsorbed carbonate species, while the N_2_-sorption isotherms suggested a mesoporous and macroporous structure, which is advantageous for dye adsorption and subsequent degradation. The BET surface area of 52 m^2^/g supports the observed high photocatalytic activity.

The photocatalytic studies demonstrated that MgO nanoparticles exhibit excellent degradation capabilities for both RhB and R6G dyes. Under UV light, MgO achieved complete degradation of RhB within 180 min, while under visible light, it achieved 83.23% degradation. For R6G, the degradation efficiency was 92.62% under UV light and 38.71% under visible light, indicating a higher photocatalytic activity towards RhB under visible light. These results highlight the potential of MgO nanoparticles as effective photocatalysts for the degradation of organic dyes in wastewater.

The results align well with previous studies that have highlighted the effectiveness of metal oxide nanoparticles in photocatalytic applications. For instance, TiO_2_ and ZnO have been widely studied and reported to exhibit significant photocatalytic properties. However, MgO offers several advantages, including non-toxicity, biocompatibility, and a lower band gap, which enhances its activity under visible light.

The photocatalytic mechanism proposed in this study is consistent with the general principles observed in other semiconductor photocatalysts. Electron-hole pairs’ generation upon light irradiation and the subsequent production of ROS like •OH radicals play a crucial role in dyes’ degradation. The scavenging experiments confirmed that •OH radicals are the dominant species in the degradation process for both RhB and R6G, similar to findings in studies involving TiO_2_ and ZnO photocatalysts.

The findings of this study have significant implications for wastewater treatment, particularly in industries that discharge dye-contaminated effluents. The high photocatalytic efficiency of MgO nanoparticles under both UV and visible light suggests their potential application in real-world scenarios, where visible light comprises a major portion of the solar spectrum. This could lead to more sustainable and cost-effective wastewater treatment processes. Comparing the results obtained from previous studies, that had focused on the use of well-established photocatalysts, such as pure TiO_2_ and ZnO, it is clear that MgO powder could totally degrade rhodamine B upon a 3 h UV light irradiation, while ZnO [98] and TiO_2_ nanoparticles [99] needed less than 3 h for the same effect. Under visible light irradiation, MgO powder led to 83.23% degradation of rhodamine B, while ZnO nanoparticles could totally degrade rhodamine B in the same timeframe [98]. TiO_2_ is not so efficient under visible light irradiation (TiO_2_ Evonik P25 can degrade rhodamine B by 48% after 240 min) [100], and this is why it is widely doped for the enhancement of its photocatalytic performance under visible light irradiation. Regarding rhodamine 6G, MgO achieved 92.62% and 38.71% degradation after 3 h of UV and visible light illumination, respectively. According to Pino et al., when UV or visible light is applied for 90 min to irradiate a solution of rhodamine 6G in the presence of TiO_2_ Evonik P25, a degradation percentage of 22% is determined [14]. MgO led to a 35% degradation of rhodamine 6G after 90 min of visible light irradiation and ~70% under UV light irradiation, thus MgO is proven as an efficient photocatalyst of rhodamine 6G. ZnO degraded by 72% rhodamine 6G, under UV light irradiation for 120 min, according to Yudasari et al. [101]. In the same timeframe, MgO degraded by >85% rhodamine 6G. Also, according to Khoza et al., ZnO composites led to a 50% degradation of rhodamine 6G after 60 min of photoactivation with visible light, showing also excellent reusability after five cycles [102]. So, MgO seems to be a very promising photocatalyst against rhodamine B or rhodamine 6G, compared to TiO_2_ and ZnO.

Furthermore, the study highlights the importance of nanoparticle size, surface area, and the presence of active sites in enhancing photocatalytic activity. These insights can guide the design and synthesis of more efficient photocatalysts in the future.

Future research could focus on optimizing the synthesis process to further reduce the particle size and increase the surface area of MgO nanoparticles, thereby enhancing their photocatalytic efficiency. Additionally, exploring the doping of MgO with other metal ions could improve visible light’s absorption and enhance the generation of ROS.

Investigating the reusability and stability of MgO nanoparticles in long-term photocatalytic applications is also crucial. While this study demonstrated significant photostability over five cycles, extended research is required to grasp the mechanisms behind any noted deactivation and to develop strategies for regeneration.

Finally, extending the study to other types of organic pollutants and exploring the photocatalytic performance of MgO in real wastewater samples would provide a more comprehensive understanding of its potential applications in environmental remediation.

In conclusion, this study underscores the potential of MgO nanoparticles as efficient photocatalysts towards organic dye degradation, paving the way for their application in sustainable wastewater treatment technologies.

## 4. Materials and Methods

### 4.1. Synthesis of MgO Powder

**Figure 18:**
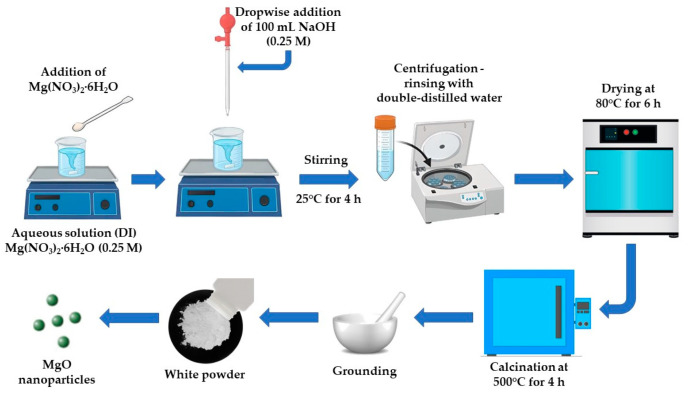
Schematic illustration for the synthetic procedure of MgO powder.

The synthesis of MgO powder was conducted utilizing a facile precipitation approach, founded on the synthetic protocol of Karthikeyan and colleagues [103], upon some alterations. In particular, 6.4103 g of magnesium nitrate hexahydrate (Mg(NO_3_)_2_•6H_2_O, 99%, Sigma-Aldrich, Darmstadt, Germany) were added in 100 mL of lab-distilled water. Subsequently, 100 mL of a 0.25 M sodium hydroxide solution (NaOH, 99.5%, Panreac Quimica SA, Barcelona, Spain) were poured dropwise into the aforementioned aqueous solution. The acquired mixture underwent continuous magnetic stirring for 4 h at 25 °C until the emergence of a white-colored suspension. The completion of the reaction procedure was indicated by the formation of a white precipitate, which was acquired through centrifugation and was subsequently triturated and purified via rinsing with double-distilled (18.2 MΩ·cm) water and centrifugation for eliminating potential impurities. Then, the obtained precipitate underwent drying at 80 °C for 6 h and was further calcinated at 500 °C (4 h), finally resulting in a white powder’s production (Figure 18). The reaction that took place during the synthetic procedure is outlined through the following equation (Equation (19)):

(19) $$Mg{{(NO}_{3})}_{2}+NaOH\to {Mg(OH)}_{2}↓+Na{NO}_{3}+H_{2}O$$

Mg(OH)_2_ formed by the reaction of Mg(NO_3_)_2_ with NaOH when calcinated at 500 °C for 4 h results in the formation of MgO (Equation (20)) [104]:

(20) $${Mg(OH)}_{2}\to MgO+H_{2}O$$

### 4.2. Characterization of MgO Nanopowder

FESEM analysis was utilized in order to assess the morphology of the MgO powder (FESEM, JSM-7401F, JEOL, Tokyo, Japan).

Regarding the XRD analysis, a Brucker D8 Advance (Brucker, Karlsruhe, Germany) X-ray diffractometer was utilized, implementing CuΚα radiation (λ = 1.5406 Å) (40 kV, 40 mA). The measurements were conducted at a 2-theta angle ranging from 20° to 90° (0.01°/1.0 s).

FTIR measurements were also performed, and spectra were acquired at 25 °C in the range from 400 cm^−1^ to 4000 cm^−1^ (resolution: 4 cm^−1^) through a FTIR JASCO4200 apparatus (Oklahoma City, OK, USA), possessing a Ge crystal.

The synthesized powder’s N_2_ adsorption was examined via a ChemBET 3000 instrument (Yumpu, Diepoldsau, Switzerland) to ascertain the BET specific area. Before each measurement, the MgO powder passed through a degassing process (80 °C, 24 h).

Thermogravimetric analysis was performed utilizing a Mettler Toledo TGA/DSC 1 HT apparatus (Mettler Toledo GmbH, Greifensee, Switzerland). Measurements were conducted under N_2_ flow (10 mL/min) in the range 30–1000 °C and a heating rate equal to 10 °C/min.

XPS analysis (Leybold SPECS LHS/EA10, Leybold GmbH, Cologne, Germany) was implemented in order to assess the examined powder’s surficial chemical states. An ultra-high vacuum chamber (P ≈ 5 × 10^−10^ mbar) equipped with a SPECS Phoibos 100 hemispherical electron analyzer (Berlin, Germany) with a delay line detector (DLD) and an unmonochromized dual-anode Mg/Al X-ray source were utilized for the measurements. A MgKα line at 1253.6 eV and an analyzer pass energy of 10 eV (giving a FWHM equal to 0.85 eV for the Ag 3d_5/2_ peak) were utilized. A fitting routine was used for analyzing the XPS core level spectra, leading to each spectrum’s decomposition into individual mixed Gaussian–Lorentzian peaks upon a Shirley background subtraction. Errors regarding peak areas were found equal to ≈10%, and the accuracy for binding energies’ assignments was approximately 0.1 eV. The samples, which were originally in powder form, were compressed into pellets for measurement. Analysis was conducted on a 3 mm diameter area, with the XPS spectra documented at 25 °C.

The hydrodynamic diameter, as well as the distribution of the powder’s particles in an aqueous dispersion, was assessed through dynamic light scattering (DLS) (Malvern Zetasizer Nano ZS, Malvern Panalytical Ltd., Malvern, UK). The scattering intensity’s recording was achieved using a 633 nm laser and a 173° scattering angle.

Diffuse reflectance measurements for obtaining the E_g_ values were evaluated via a UV–vis spectrometer (Jasco UV/Vis/NIR V-770, Interlab, Athens, Greece) possessing an integrating sphere.

RhB’s and R6G’s mineralization percentage was evaluated by TOC analysis (TOC-LCSH/CSN, Shimadzu Scientific Instruments, Columbia, MD, USA).

### 4.3. Photocatalytic Efficiency Study of MgO Nanopowder

The photocatalytic effectiveness of the as-prepared MgO powder upon both UV and visible light irradiation was initially evaluated towards the degradation of rhodamine B through the addition of 0.005 g of the powder in a 10 ppm aqueous solution (250 mL) of RhB (C_28_H_31_CIN_2_O_3_, ≥95%, Penta-Chemicals Unlimited, Prague, Czech Republic) at 25 °C and pH value equal to 6.71 ± 0.01. Before each photocatalytic experiment, the rhodamine B solution was saturated for 1 h via extra-pure O_2_ (99.999%) flow.

In addition, the assessment of the MgO’s photocatalytic activity was conducted under UV and visible light illumination towards rhodamine 6G (C_28_H_31_N_2_O_3_Cl, 99%, Sigma-Aldrich, Darmstadt, Germany) degradation, using the same conditions as described in the case of rhodamine B with the only difference that the pH value of the dye’s solution was equal to 7.48 ± 0.01.

The photoreactor that was utilized for the photocatalytic experiments was equipped with four parallel lamps placed 10 cm above each sample’s surface [78]. Blacklight lamps (368 nm, 830 lumens, incident light flux: 0.184 μmol quanta/s, Sylvania, Wilmington, NC, USA) were employed as the UV irradiation source, while 15 W visible light lamps (900 lumens, 400 nm cutoff filter, incident light flux: 0.371 μmol quanta/s, OSRAM GmbH, Munich, Germany) comprised the visible light irradiation source. All the experiments were conducted at 25 °C [53,78].

The derived absorbance of the studied MgO powder was estimated at 554 nm [53] and 525 nm [105] for RhB and R6G, respectively, utilizing a spectrometer (Thermo Fisher Scientific Evolution 200, Thermo Fisher Scientific, Waltham, MA, USA). The C/C_0_ ratio, where C is ascribed to RhB’s and R6G’s concentration after a certain time of photocatalysis and C_0_ corresponds to RhB and R6G initial concentration, was acquired indirectly by the evaluation of the measured absorption A (absorption at each time) to the initial absorption (A_initial_) [78].

## 5. Conclusions

In this study, magnesium oxide (MgO) nanoparticles were synthesized using a simple precipitation method and characterized by various techniques, confirming their high purity, crystallinity, and appropriate physicochemical properties for photocatalytic applications. The MgO nanoparticles demonstrated significant photocatalytic efficiency in degrading rhodamine B (RhB) and rhodamine 6G (R6G) dyes under both UV and visible light irradiation. The nanoparticles exhibited complete degradation of RhB under UV light within 180 min and achieved notable degradation levels for R6G as well. The study’s findings underscore the potential of MgO nanoparticles as a promising photocatalyst, particularly for the selective degradation of hazardous dyes such as RhB, thereby contributing to the development of more effective wastewater treatment technologies. Additionally, the reusability of MgO nanoparticles across multiple trials further emphasizes their practical applicability, making them a viable candidate for large-scale environmental remediation efforts. Future research could focus on optimizing the synthesis process to enhance the photocatalytic performance of MgO nanoparticles under visible light and exploring their efficacy in degrading other persistent organic pollutants.

## Figures and Tables

**Table 1:** Comparison of various synthetic approaches towards MgO nanoparticles’ fabrication, regarding the average crystallite size and specific surface area.

Synthetic Approach	Average Crystallite Size (nm)	Specific Surface Area (m^2^/g)	Reference
Sol–gel	12–13	-	[42]
Microwave-assisted sol–gel	9.5–10.5	243.2	[43]
Ultrasonic-assisted sol–gel	19.2	-	[44]
Modified thermal/sol–gel	23.6	257.3	[45]
Solid-state chemical	10.5	213	[40]
Microwave irradiation	16	70	[46]
Precipitation	25	216.9	[47]

**Table 2:** Crystal lattice indices, average crystallite size, FWHM (Full Width at Half Maximum), and crystallinity index of the synthesized MgO powder.

Sample ID	Crystal Lattice Index (*a* = *b* = *c*)			Average Crystallite Size (D, nm) *	FWHM	CI (%)
	*a*	*b*	*c*			
MgO	4.2194	4.2194	4.2194	3.23	0.4562	80.49

* The (200) plane’s peak was used to estimate the crystallite size.

**Table 3:** d-Spacing calculations for MgO powder.

Bragg’s Angle		d_hkl_ (Å)	d_hkl_ (nm)	hkl
2θ	θ			
36.85	18.43	2.4372	0.2437	111
42.83	21.42	2.1097	0.2110	200
61.20	30.60	1.5132	0.1513	220
74.58	37.29	1.2714	0.1271	311
78.51	39.26	1.2173	0.1217	222

**Table 4:** Data obtained via the BET approach. (a) Specific surface area estimated utilizing Brunauer–Emmett–Teller theory, (b) micropore surface area through t-plot analysis, based on the Harkins and Jura model, (c) cumulative volume of pores in the range 1.7 and 300 nm from N_2_-sorption data and the BJH desorption approach, and (d) average pore diameter, evaluated by the 4 V/σ approach (V was equated to the maximum volume of N_2_ adsorbed along the isotherm as P/P_o_ → 1.0).

Sample ID	BET Surface Area (m^2^/g)	Micropore Surface Area (m^2^/g)	Cumulative Pore Volume (cm^3^/g)	Average Pore Diameter (nm)
MgO	52	2	0.3	21

**Table 5:** Size distribution and zeta potential data acquired from DLS measurements utilizing an aqueous dispersion solution of the examined MgO powder.

Sample ID	Hydrodynamic Diameter (D_h_) (nm)	Zeta Potential (mV)	PDI
MgO	27.11 ± 0.93	−50.8 ± 0.6	0.197 ± 0.093

**Table 6:** Kinetic parameters of the studied powder upon UV and visible light photocatalytic trials.

Sample ID	Pseudo-First-Order Kinetic Model		Pseudo-Second-Order Kinetic Model	
	k_1_ (min^−1^)	R^2^	k_2_ (g/mg·min)	R^2^
MgO (visible)	0.002	0.974	10.747	0.778
MgO (UV)	0.012	0.982	5.545	0.906

**Table 7:** Kinetic parameters of the studied powder upon UV and visible light photocatalysis towards degradation of R6G dye.

Sample ID	Pseudo-First-Order Kinetic Model		Pseudo-Second-Order Kinetic Model	
	k_1_ (min^−1^)	R^2^	k_2_ (g/mg·min)	R^2^
MgO (visible)	0.002	0.969	1.772	0.911
MgO (UV)	0.014	0.981	0.756	0.907

## Data Availability

Data are contained within the article and Supplementary Materials.

## Supplementary Materials

The following supporting information can be downloaded at: https://www.mdpi.com/article/10.3390/molecules29184299/s1: Figure S1: Nelson–Riley plot of the synthesized MgO powder; Figure S2: Williamsons-Hall (W-H) plot of the as-studied MgO powder; Figure S3: (a) Mg2p peak and (b) XAES detailed region of Mg KLL of the MgO powder; Figure S4: (a) Size distribution diagram and (b) zeta potential diagram of the as-synthesized MgO powder; Figure S5: (a) F(R) reflectance plotted against wavelength for the MgO powder under study and (b) the E_g_ of the same powder; Figure S6: Real-time UV–visible spectra obtained upon (a) UV and (b) visible light induced photocatalytic degradation of RhB utilizing the as-synthesized MgO powder; Figure S7: Photocatalytic kinetic model studies for the studied MgO powder, following a pseudo-second-order model upon UV and visible light illumination; Figure S8: XRD patterns of MgO photocatalyst after the reusability studies; Figure S9: Real-time UV–visible spectra obtained upon (a) UV and (b) visible light induced photocatalytic degradation of R6G utilizing the as-synthesized MgO powder; Figure S10: Photocatalytic kinetic model studies for the examined MgO powder, following a pseudo-second-order model upon UV and visible light illumination.
