# Supplementary material for: MgO Nanoparticles as a Promising Photocatalyst towards Rhodamine B and Rhodamine 6G Degradation
Source: Molecules. 2024 Sep 11;29(18):4299. doi: 10.3390/molecules29184299 (PMC11434436; doi:10.3390/molecules29184299)
Supplement: Supplementary file 1 [file molecules-29-04299-s001.zip › molecules-3101978-supplementary.pdf]

SUPPLEMENTARY MATERIAL

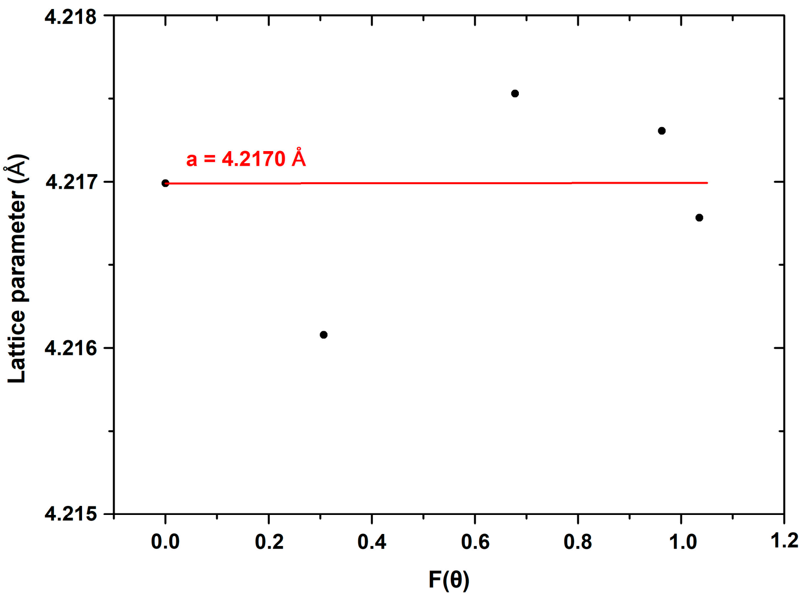

Figure S1. Nelson–Riley plot of the synthesized MgO powder.

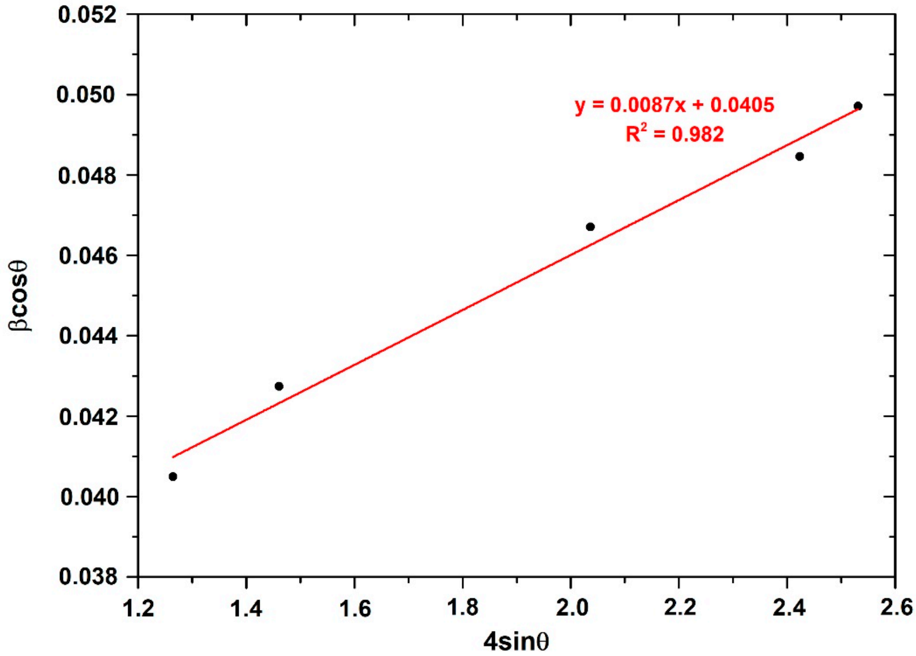

Figure S2. Williamsons-Hall (W-H) plot of the as-studied MgO powder.

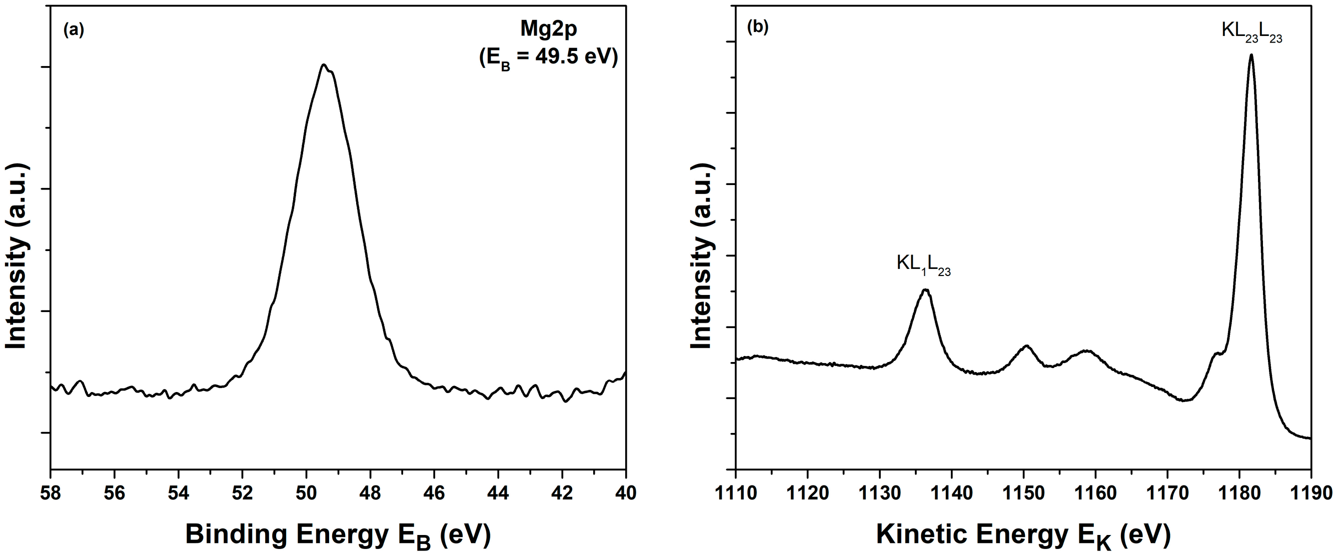

Figure S3. (a) Mg2p peak and (b) XAES detailed region of Mg KLL of the MgO powder.

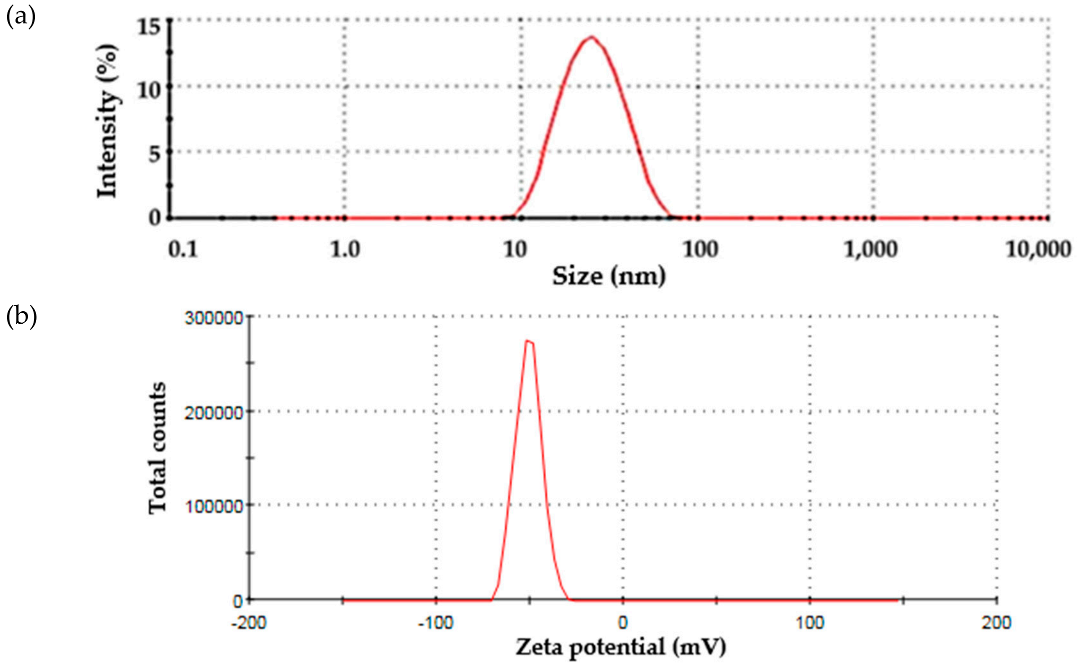

Figure S4. (a) Size distribution diagram and (b) zeta potential diagram of the as-synthesized MgO powder.

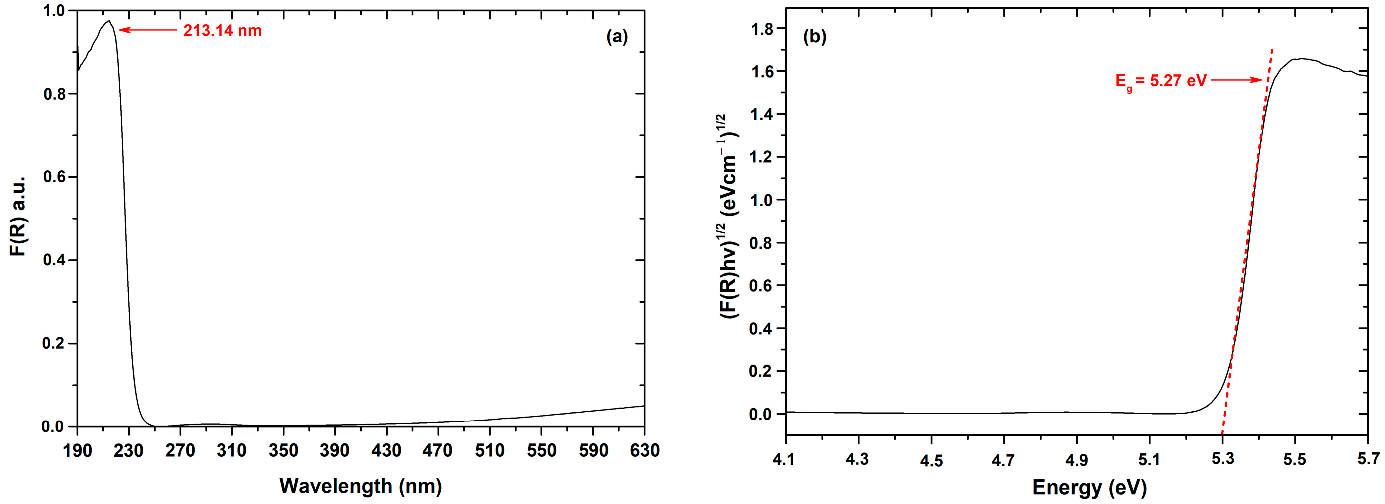

Figure S5. (a) F(R) reflectance plotted against wavelength for the MgO powder under study and (b) the E_g of the same powder.

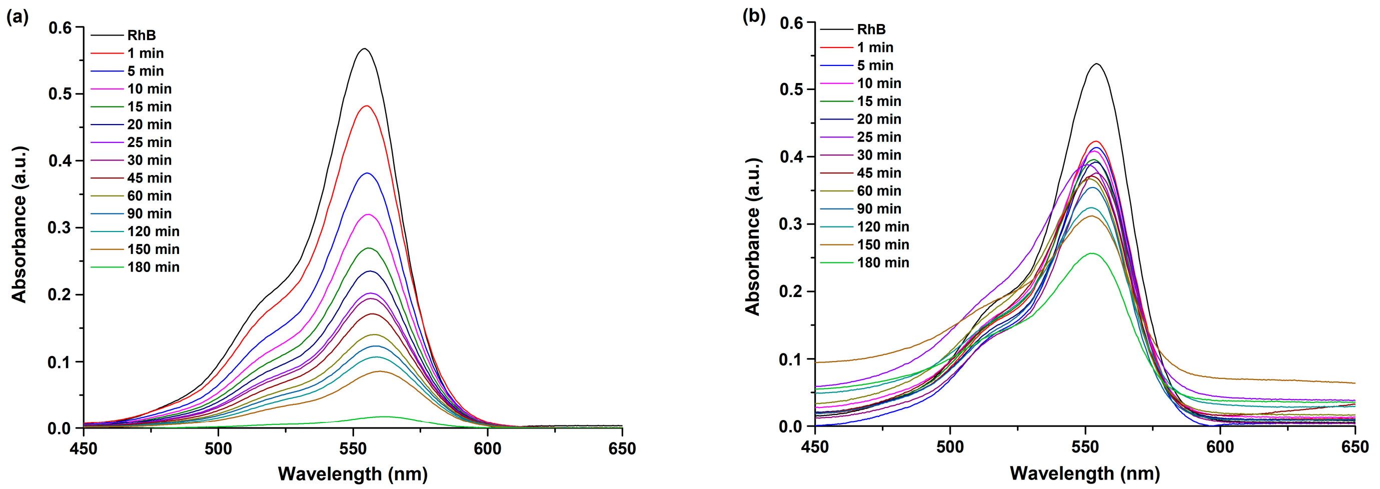

Figure S6. Real-time UV-visible spectra obtained upon (a) UV and (b) visible light induced photocatalytic degradation of RhB utilizing the as-synthesized MgO powder.

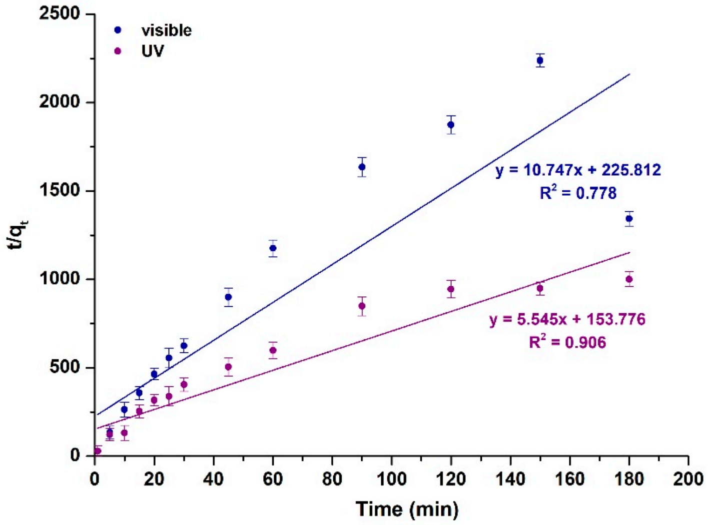

Figure S7. Photocatalytic kinetic model studies for the studied MgO powder, following a pseudo-second-order model upon UV and visible light illumination.

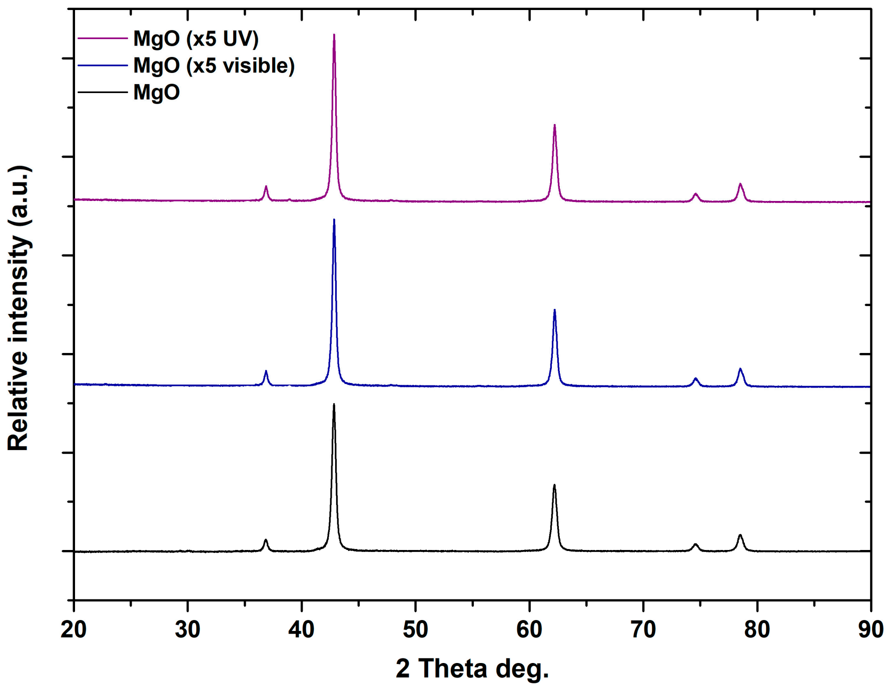

Figure S8. XRD patterns of MgO photocatalyst after the reusability studies.

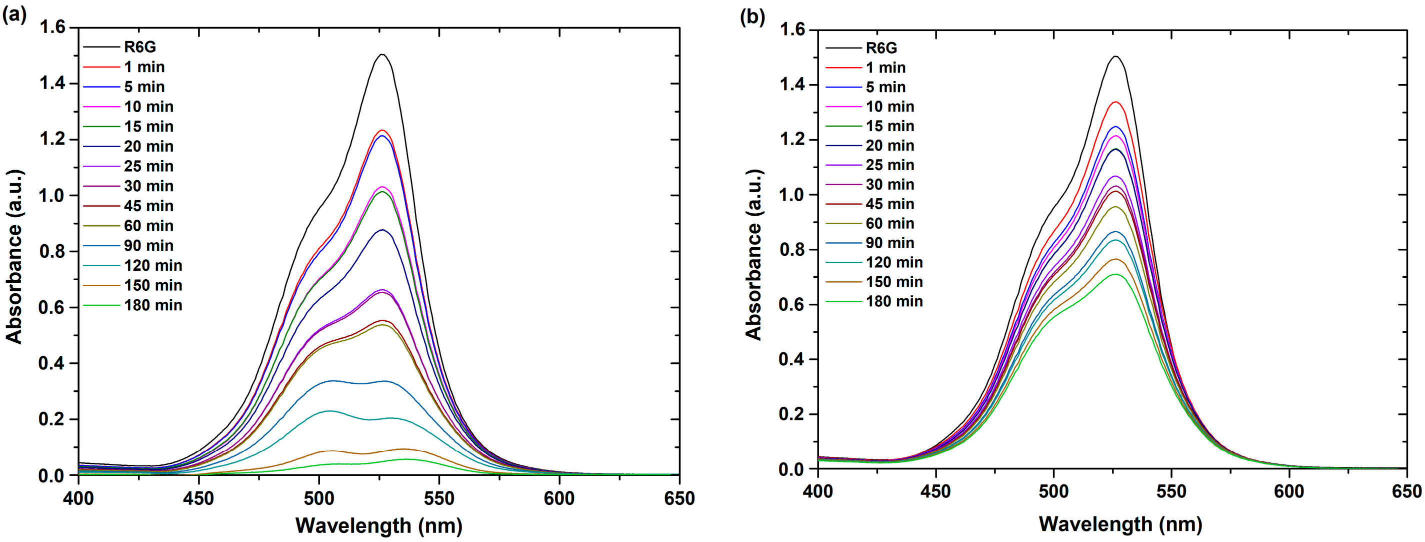

Figure S9. Real-time UV-visible spectra obtained upon (a) UV and (b) visible light induced photocatalytic degradation of R6G utilizing the as-synthesized MgO powder.

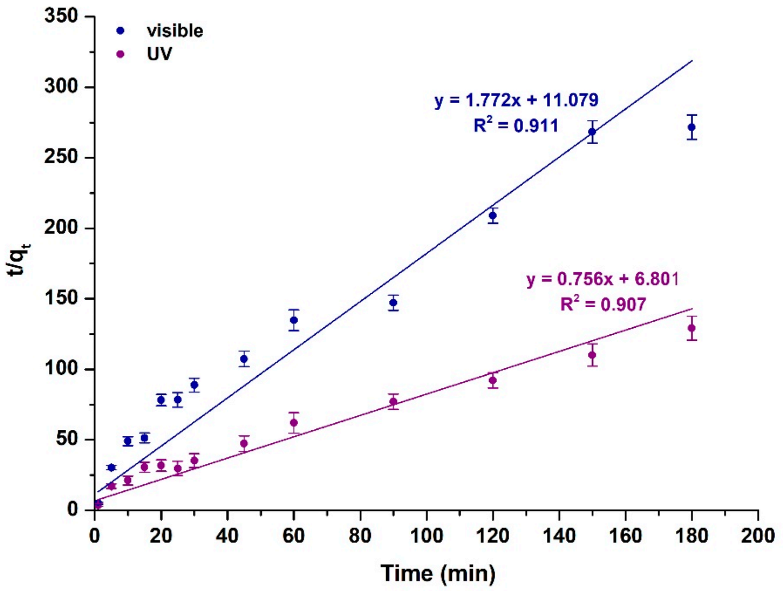

Figure S10. Photocatalytic kinetic model studies for the examined MgO powder, following a pseudo-second-order model upon UV and visible light illumination.
